# Cross-Modal Coordination of Face-Directed Gaze and Emotional Speech Production in School-Aged Children and Adolescents with ASD

**DOI:** 10.1038/s41598-019-54587-z

**Published:** 2019-12-04

**Authors:** Tanner Sorensen, Emily Zane, Tiantian Feng, Shrikanth Narayanan, Ruth Grossman

**Affiliations:** 1 Signal Analysis and Interpretation Laboratory, Department of Electrical and Computer Engineering, University of Southern California, Los Angeles, 90089 USA; 2 Department of Communication Sciences and Disorders, Emerson College, Boston, MA 02116 USA

**Keywords:** Autism spectrum disorders, Emotion, Human behaviour

## Abstract

Autism spectrum disorder involves persistent difficulties in social communication. Although these difficulties affect both verbal and nonverbal communication, there are no quantitative behavioral studies to date investigating the cross-modal coordination of verbal and nonverbal communication in autism. The objective of the present study was to characterize the dynamic relation between speech production and facial expression in children with autism and to establish how face-directed gaze modulates this cross-modal coordination. In a dynamic mimicry task, experiment participants watched and repeated neutral and emotional spoken sentences with accompanying facial expressions. Analysis of audio and motion capture data quantified cross-modal coordination between simultaneous speech production and facial expression. Whereas neurotypical children produced emotional sentences with strong cross-modal coordination and produced neutral sentences with weak cross-modal coordination, autistic children produced similar levels of cross-modal coordination for both neutral and emotional sentences. An eyetracking analysis revealed that cross-modal coordination of speech production and facial expression was greater when the neurotypical child spent more time looking at the face, but weaker when the autistic child spent more time looking at the face. In sum, social communication difficulties in autism spectrum disorder may involve deficits in cross-modal coordination. This finding may inform how autistic individuals are perceived in their daily conversations.

## Introduction

A core diagnostic criterion for autism spectrum disorder (ASD) is persistent difficulties in social communication and social interaction affecting both verbal and nonverbal communication^1^. The fastest growing group of individuals on the autism spectrum have language and cognitive abilities within the normal range, but continue to exhibit atypical verbal expressions and non-verbal facial expressions that often lead them to be perceived as awkward by others^2–4^, including age-matched peers with and without ASD^5,6^.

Prior work has shown that emotional speech productions of children with ASD are rated as more emotionally intense, but also as more awkward than those of their NT peers^3,7^. The perceived awkwardness in speech production of individuals with ASD may have its basis in properties such as increased pitch range^8^ (especially in emotional sentences^5^), variable loudness and voice quality^9,10^, atypical rate^11^, and overall differences in prosodic contours^12^. There is also evidence to suggest that individuals with ASD have a level of imbalance across these acoustic features that significantly differentiates their speech production from those of their neurotypical (NT) peers^13^. Independent raters found autistic speech production to be more awkward or “odd” than that of NT participants^2,7^.

Research exploring facial expression in ASD is relatively limited, but dynamic motion capture analyses may explain the perceived awkwardness of facial expressions in individuals with ASD. Evidence includes greater expressive ambiguity in children with ASD, such that the emotional valence of target expressions (i.e., positive vs. negative) does not predict facial movement patterns. In contrast, emotional valence has strong predictive power for facial movements in NT children^14^. Children with ASD also exhibit atypical timing and synchrony of movements of different facial regions^15^, reduced intensity of upper face movement^16^, and a reduced variety of facial movements^17^.

There is a richer literature on facial processing in ASD than on facial expression in ASD. The literature on facial processing deals in particular with visual attention to faces. Research findings in this area vary greatly, with some studies documenting reduced gaze to faces^18–21^ and others reporting gaze patterns to faces that are not different from those of NT peers^22–24^. A review of the literature does not support the hypothesis that autistic individuals gaze disproportionately at the mouth while avoiding the eyes^25^, but the majority of studies do document that individuals with ASD show reduced gaze to the face compared to their NT peers^26^. It is reasonable to propose that this reduced face-directed gaze would provide individuals with ASD fewer opportunities to perceive and process social communicative facial expressions, thereby potentially contributing to the documented atypical production of communicative expressions and reduced social skills overall^18^.

In addition to showing divergent patterns in facial expression, speech production, and face-directed gaze, individuals with ASD have been shown to have difficulty integrating information across modalities. During receptive tasks, autistic individuals struggle with cross-modal integration^27^, particularly in relation to information from faces and voices^28,29^. In addition to these receptive deficits, there is some evidence that individuals with ASD struggle with cross-modal integration during expressive tasks. For instance, when individuals with ASD tell a narrative, their gestures are less coordinated with the timing of speech production than those of NT individuals^30^.

Despite this evidence of receptive difficulties in cross-modal integration, there have been no quantitative behavioral studies to date on how autistic individuals coordinate vocal and facial expressions during speech production and whether the presence of atypical face-directed gaze is related to any differences in vocal and facial expression quality or coordination. Such information is crucial to understanding the relation between receptive and expressive social communication skills and crucial to documenting possible reasons for the perceived awkwardness of facial and vocal expressions of autistic individuals^4^.

The objective of the present study is to analyze the dynamic relation between speech production and facial movement and to establish how face-directed gaze modulates this cross-modal coordination of expressive modalities in NT and autistic children. We presented a dynamic mimicry task using video stimuli of neutral and emotional sentences spoken by several actors. We recorded audio, video, motion capture, and eyetracking data from a group of autistic and NT children as they watched and mimicked each stimulus. We used Granger causality analysis to quantify cross-modal coordination between speech production and facial movement. Granger causality is a measure of how well a signal $y$ is predicted by another signal $x$^31^. Specifically, Granger causality indicates whether using past values of a signal $x$ to predict a signal $y$ improves prediction beyond what can be achieved using only past values of $y$. For the purpose of the present study, if movements of the face strongly Granger-cause the speech signal, this indicates strong cross-modal coordination between speech production and facial movements, showing that the face is moving synchronously with speech production. Weak Granger causality, on the other hand, indicates weak cross-modal coordination, indicating that the face moves asynchronously with speech production. The dependent variable of the present study, “cross-modal coordination”, is defined as Granger causality strength.

Granger causality analysis determines causality through analysis of the statistical dependencies between two signals, not through direct experimental manipulation of one or the other signal. Such an analysis is useful for human behavior, where direct experimental control is frequently either infeasible or entirely unavailable. For this reason, Granger causality has been used for the analysis of facial movement in behavioral autism research^15–17^. Whereas previous studies use Granger causality to establish whether an individual coordinates the expressions of different facial regions, the present study proposes a new application of this method, namely, to establish whether an individual coordinates facial expression with speech production.

Based on existing evidence that individuals with ASD display perceptual deficits in cross-modal integration and reduced synchrony of facial movements, we predict that children with ASD will display weaker cross-modal coordination between facial expression and speech production than NT children, particularly for emotional sentences that require greater facial and vocal expressiveness. Given the evidence of reduced face-directed gaze in ASD, we also predict that increased gaze to the stimulus faces will be associated with the NT pattern of strong cross-modal coordination.

## Results

### Effect of diagnosis on cross-modal coordination

We performed statistical tests of linear mixed effects model coefficients to test for an effect on cross-modal coordination for factor *diagnosis* (treatment: ASD; baseline: NT), factor *sentence* (treatment: emotional; baseline: neutral), and covariate *age*. The interactions were the following: *diagnosis* × *sentence*; *age* × *diagnosis*; *age* × *sentence*; *age* × *diagnosis* × *sentence*.

#### Diagnosis, sentence, and their interaction

**Figure 1:**
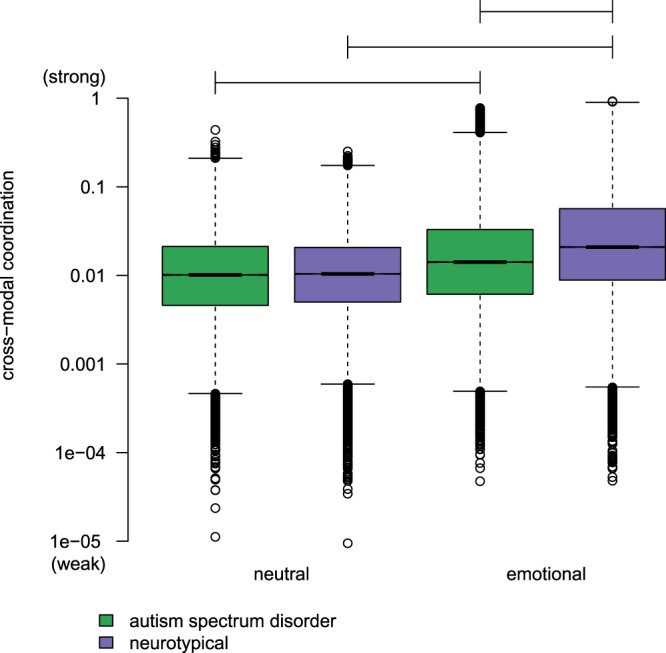
Distribution of cross-modal coordination during the emotional and neutral sentences for the ASD group (green) and NT group (purple). Horizontal lines indicate significant differences in expected marginal means. Comparisons are between levels of factor *diagnosis* with the level of factor *sentence* fixed and between levels of factor *sentence* with the level of factor *diagnosis* fixed. Y-axis scale is logarithmic.

The factor *diagnosis* did not have a significant effect on cross-modal coordination ($t(31.2)=-\,0.77$, $p=0.45$), but the factor *sentence* was significant ($t(2.98\times 1{0}^{5})=42.8$, $p < 1\times 1{0}^{-16}$) and the interaction of *diagnosis* and *sentence* was significant ($t(2.98\times 1{0}^{5})=-\,15.7$, $p < 1\times 1{0}^{-16}$). We explored these effects with a simple effects analysis in which we compared the expected marginal means for factor *diagnosis* at each level of factor *sentence* and, vice versa, the expected marginal means for factor *sentence* at each level of factor *diagnosis* (see Fig. 1). Consistent with the main effect for *sentence*, we found that mean cross-modal coordination differed significantly between neutral and emotional sentences, with emotional sentences displaying stronger cross-modal coordination than neutral sentences (for children with ASD: $t(3.0\times 1{0}^{5})=67.92$, $p < 1\times 1{0}^{-16}$; for NT children: $t(3.0\times 1{0}^{5})=169.60$, $p < 1\times 1{0}^{-16}$). Consistent with the *diagnosis* × *sentence* interaction, we found that NT children had significantly stronger cross-modal coordination in emotional sentences than children with ASD ($t(31.3)=-\,6.40$, $p=3.8\times 1{0}^{-7}$), but in neutral sentences we found no group difference ($t(31.3)=-\,2.02$, $p=0.052$).

These results indicate that emotional sentences have stronger cross-modal coordination than neutral sentences and that the difference between emotional and neutral sentences is greater for NT children than for children with ASD. NT children produce emotional sentences with strong cross-modal coordination and produce neutral sentences with weak cross-modal coordination. Children with ASD, however, produce similar levels of cross-modal coordination for neutral and emotional sentences.

We conducted a follow-up analysis to determine whether this finding was the result of children with ASD being less emotionally expressive in facial expression and speech production and therefore producing cross-modal coordination patterns for emotional phrases that were similar to those for neutral phrases. We used motion capture marker variance (i.e., a measure of facial expression intensity) and audio feature variance (i.e., a measure of speech production intensity) to investigate potential diagnosis-based differences in overall facial expressiveness. We controlled for individual variability such as head size by estimating by-participant random intercepts as part of a linear mixed effects model. The by-participant random intercepts accounted for random variation at the level of study participant, while still allowing us to evaluate the fixed effect for diagnosis (contrast: $\,{\rm{ASD}}-{\rm{NT}}\,$). We also analyzed overall intensity of acoustic features to account for vocal expressiveness. There was no significant difference in intensity between the ASD and NT groups in motion capture marker variance ($t(34.0)=2.41$, $p=0.022$) or in audio feature variance ($t(34.5)=0.04$, $p=0.97$). This indicates that children with ASD are producing emotional facial expressions and speech at similar levels of intensity, but with lower levels of cross-modal coordination for emotional expressions than their NT peers.

#### Interaction of age with diagnosis and sentence

**Figure 2:**
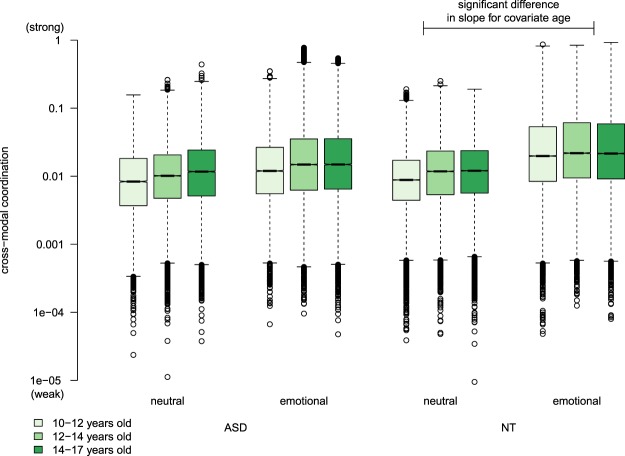
Distribution of cross-modal coordination during the emotional and neutral sentences for the ASD group and NT group for ages 10–12 years (light), 12–14 years (medium), and 14–17 years (dark). Pairwise comparisons are made between slopes for each *diagnosis* × *sentence* cell. The significant difference in slope for age is between the ASD-neutral cell and ASD-emotional cell (horizontal line). Y-axis scale is logarithmic. Abbreviations: ASD – autism spectrum disorder; NT – neurotypical.

Neither the covariate *age* ($t(31.1)=2.48$, $p=0.02$) nor its interaction with *diagnosis* was significant ($t(31.2)=0.48$, $p=0.64$). The interaction of *age* and *sentence* was significant ($t(2.98\times {10}^{5})=-16.0$, $p < 1\times 1{0}^{-16}$), indicating that cross-modal coordination in emotional sentences was stronger for younger children than for older children, irrespective of diagnosis. The three-way interaction of *age*, *diagnosis*, and *sentence* was likewise significant ($t(2.98\times 1{0}^{5})=8.70$, $p < 1\,\times \,1{0}^{-16}$). We explored these effects by performing pairwise comparisons between *diagnosis*$\,\times \,$*sentence* cells in terms of the expected marginal trends for covariate *age* (see Fig. 2). We found a significant difference in slope for *age* between NT-neutral and NT-emotional cells, with *age* having a greater effect in the NT-neutral cell than in the NT-emotional cell ($t(3.0\,\times \,1{0}^{5})=16.0$, $p < 1\,\times \,1{0}^{-16}$). This indicates that older NT children display stronger cross-modal interaction in emotional sentences than younger NT children. We do not find evidence of this trend in the ASD group.

### Face-directed gaze

**Figure 3:**
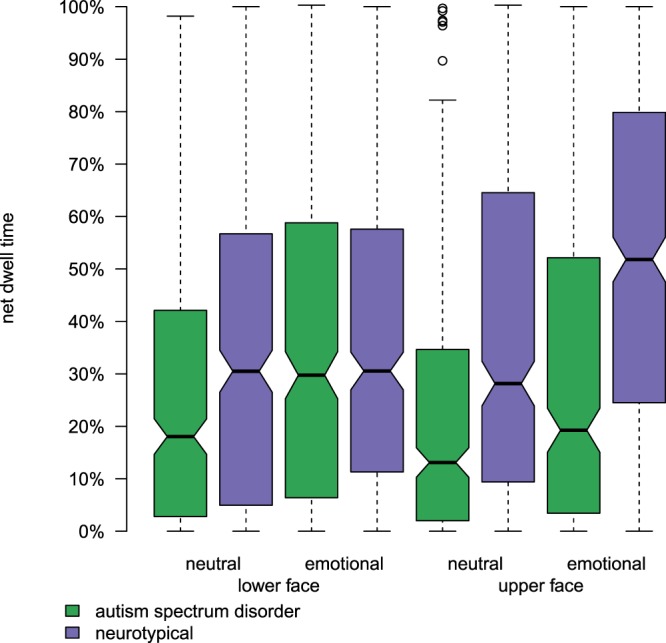
Distribution of net dwell times to the lower face and upper face during the emotional and neutral sentences for the ASD group (green) and NT group (purple). Y-axis scale is as percentage of total duration of visual stimulus.

We performed statistical tests of linear mixed effects model coefficients to test for an effect on face-directed gaze for factors *diagnosis* (contrast: $\,{\rm{ASD}}-{\rm{NT}}\,$), *sentence* (contrast: emotional − neutral), *area of interest* (contrast: $\,{\rm{upper\; face}}-{\rm{lower\; face}}\,$), and all interactions of these factors. Factor *diagnosis* was significant ($t(45)=-\,3.610$, $p=7.7\times 1{0}^{-4}$, indicating that mean gaze duration is significantly greater for NT children ($\,{\rm{mean}}\,=\,40.0$, $\,{\rm{S.E.}}\,=\,2.3$) than for children with ASD ($\,{\rm{mean}}=28.4$, $\,{\rm{S.E.}}=2.3$). The main effect of *sentence* was significant ($t(149.2)=10.6$, $p < 1\,\times \,1{0}^{-16}$), indicating that mean gaze duration was significantly greater during the emotional sentence (${\rm{mean}}=38.5$, $\,{\rm{S.E.}}=1.6$) than during the neutral sentence (${\rm{mean}}=29.9$, $\,{\rm{S.E.}}=1.6$). The effect of *area of interest* was not significant ($t(45)=-\,0.45$, $p=0.65$). None of the interactions were significant (see Fig. 3).

### Effect of face-directed gaze on cross-modal coordination

Based on suggestions in the literature that reduced receptive experience with social cues leads to reduced expertise in expressive social cues^32^, we tested the hypothesis that increased gaze to faces leads to greater cross-modal coordination of speech production and facial expression.

Specifically, we performed statistical tests of linear mixed effects model coefficients to test for an effect on face-directed gaze for factor *diagnosis* (contrast: $\,{\rm{ASD}}-{\rm{NT}}$), covariate *uf* (upper face net dwell time), covariate *lf* (lower face net dwell time), and covariate *age*. The interactions were the following: *diagnosis* × *lf*; *diagnosis* × *uf*; *diagnosis* × *lf* × *age*; and *diagnosis* × *uf* × *age*.

The effect for covariates *lf* ($$t(2.97\times 1{0}^{5})=-\,8.17$$, $$p=3.05\times 1{0}^{-16}$$) and *uf* were significant ($$t(2.93\times 1{0}^{5})$$$$=-24.3$$, $$p=1\times 1{0}^{-16}$$). NT participants differed from ASD participants in terms of the slope for covariates *uf* ($$t(2.9\times 1{0}^{5})=-\,4.46$$, $$p=8.20\times 1{0}^{-6}$$) and *lf* ($$t(2.9\times 1{0}^{5})=-\,7.32$$, $$p=2.49\times 1{0}^{-13}$$). Children with ASD had a negative slope for gaze to the upper face (slope for *uf*: $$-0.16$$, S.E.: $$6.9\times 1{0}^{-3}$$) and for gaze to the lower face (slope for *lf*: $$-0.14$$, S.E.: $$5.3\times 1{0}^{-3}$$). NT children had a positive slope for gaze to the upper face (slope for *uf*: $$3.7\times 1{0}^{-3}$$, S.E.: $$5.0\times 1{0}^{-3}$$) and for gaze to the lower face (slope for *lf*: $$0.12$$, S.E.: $$4.5\times 1{0}^{-3}$$). Note that this analysis averaged over covariate *age* because neither the interaction of *age*, *diagnosis*, and *lf* ($$t(3.0\times 1{0}^{5})=1.05$$, $$p=0.29$$) nor the interaction of *age*, *diagnosis*, and *uf* were significant ($$t(2.9\times 1{0}^{5})=1.38$$, $$p=0.17$$).

Together, these results indicate that face-directed gaze is associated with strong cross-modal coordination in NT children but is associated with weak cross-modal coordination in children with ASD. For NT participants, the cross-modal coordination of speech production on facial expression is greater when the participant spends more time looking at the face. For ASD participants, cross-modal coordination is weaker with increased face-directed gaze.

## Discussion

In a dynamic speech mimicry task, children with ASD produce facial and vocal expressions that are less coordinated with each other than the facial and vocal expressions of their NT peers, particularly for emotional speech. The strong Granger causality we observe in the NT children indicates that when NT children produce emotional speech, the dynamic contours of facial movements are strongly coordinated with dynamic speech features such as pitch and tone. In contrast, weak causality in the ASD group suggests that their coordination of facial and vocal expressiveness is relatively asynchronous. Importantly, the biggest difference in causality between groups was for production of the emotional sentences (as opposed to the neutral ones). Coordination between face and voice increased substantially in the NT group, whereas the increase was much smaller in the ASD group, leading to a significant interaction between diagnostic group and emotional expressivity.

Prior work provides evidence that reduced cross-modal (facial-vocal) coordination has an impact on the perceived naturalness of connected speech in ASD. Narratives of children with ASD were rated as less engaging than those of NT children due to reduced synchrony of gestures with their corresponding speech part^30^. This supports our finding of increased cross-modal asynchrony in autistic speech. Our study further demonstrates that children with ASD exhibit greater cross-modal asynchrony in tasks demanding high emotional expressiveness. This task-dependent deficit of cross-modal synchrony may be related to the perceived awkwardness of this population.

With regard to the receptive behavior of face-directed gaze, our eyetracking analyses reveal diagnosis-based group differences in gaze behaviors. Children with ASD exhibited less net dwell time than NT children. We also find that children gazed longer at the face during emotional sentences than during neutral sentences, irrespective of diagnosis.

When we investigated the relation between these group-based differences in receptive gaze patterns and expressive cross-modal coordination, we discovered opposite patterns for the two groups. While increasing gaze to stimulus faces resulted in stronger cross-modal coordination for NT children, increasing gaze to stimulus faces resulted in weaker cross-modal coordination for children with ASD. Thus, when participants with ASD paid more visual attention to the face, they were worse at coordinating facial expression with speech production during the corresponding sentence mimicry. This somewhat surprising finding indicates that children with ASD do not seem to benefit from increased visual exposure to emotional speech but fits with our findings in the receptive and expressive modalities respectively: children with ASD are attempting to produce emotional expressions, but fail to do so in a canonical, well-coordinated way. As this is a purposeful mimicry task involving production of a complete sentence and not an automatic, involuntary facial mimicry task, this finding suggests that children with ASD may show deficits of voluntary mimicry behaviors in addition to deficits in automatic mimicry that has been reported in previous literature. We discuss differences between automatic and voluntary mimicry later in this section.

The increased visual exploration of the stimulus face we found in some autistic children may be a reflection of increased social interest (correlation between social communication questionnaire score and mean gaze to upper face during the emotional sentence: $$r=-\,0.29$$, $$t(45)=-\,2.02$$$$p=0.049$$), resulting in their working harder to reproduce the video model. However, their greater motivation and greater effort seems to actually result in less successful face-voice coordination, potentially in the same way autistic children show greater difficulty producing well-coordinated facial and vocal speech with more emotional expressivity. This interpretation resonates with previous findings that children with ASD are both more expressive and more awkward in emotional language mimicry, indicating that autistic individuals may be eager to portray emotional expressivity, but lack the ability to express those emotions in a canonical way^7^. It is possible that participants in our study who had higher levels of social motivation, as evidenced by increased face-directed gaze, consciously attempted to produce emotionally expressive speech that was beyond their cross-modal coordination ability, thereby resulting in greater face-voice asynchrony. By contrast, NT children who demonstrated more face-directed gaze were also more successful at coordinating their faces with their voices, indicating that increased social interest and face-voice synchrony were related in this population. These data are preliminary but do indicate greater cross-modal asynchrony in emotional speech productions of school-aged children and adolescents with ASD and that interventions targeted at increased face-directed gaze in ASD may not necessarily have the intended downstream effect of improved social communication quality. Further studies are necessary to determine the causal relations between social gaze, social production, and social perception.

There is an ongoing debate about whether individuals with ASD are relatively poor at mimicking others’ facial expressions and whether a dysfunctional mirror neuron system might play a role in this^33^. The majority of investigations into the mirror neuron system in ASD focused on neuroimaging techniques to identify neural networks underlying this function or facial electromyography (EMG) to capture minute and involuntary muscle movements that occur when observing others’ facial expressions^34^. The studies reviewed seem to indicate that the primary deficit in ASD related to mirror neurons is found during action observation, rather than action execution^34^. Similarly, a seminal study on facial expression production in ASD used facial EMG to show that individuals with ASD exhibit deficits in automatic, but not voluntary mimicry of facial expressions^35^. Another recent review of the literature pointed to a complex mechanism for facial expression mimicry in ASD that relies on multiple factors, including social understanding, motor mimicry, and interpretation of emotion from faces^36^. The authors state that mirror neurons may play a role particularly in automatic, subconscious mimicry of facial expressions, but are less defining for the ability to voluntarily and purposefully mimic facial expressions^36^. Given that the task presented here involved repetition of sentences with associated emotional facial expressions and that we measured expressions using motion capture, which is not sensitive enough to capture the minute, involuntary muscle movements recorded by facial EMG when participants observe others’ facial expressions, we posit that the mirror neuron system plays a less significant role in the resulting expressions.

The limitations of the study are the following. First, the study relies on a purposeful mimicry task, rather than a more natural paradigm, such as spontaneous speech or social interaction, during which spontaneous facial and vocal behaviors could be elicited. For example, rather than mimicking facial expressions and sentences, the participant could be asked to watch and respond to emotionally evocative videos^37^ or to produce speech and facial expressions in response to an imagined situation. Future work may seek to determine whether the present findings generalize to such experimental paradigms or even to observational data, which will indicate whether the findings generalize to spontaneous responses and whether cross-modal coordination differs between mimicked and spontaneous responses. Future studies should also endeavor to analyze these patterns in a larger sample to allow for generalizability of findings.

Second, the accuracy of sentence imitation was not evaluated. It is possible that accuracy in sentence imitation task performance systematically varies with cross-modal coordination and that there are not only group differences in cross-modal coordination, but also group differences in accuracy. However, the present study did not evaluate the relation between these two variables or test for group differences in sentence imitation task accuracy.

Third, the present study did not connect the observed speech production, facial expression, and eye-gaze data to human perception of expressivity or awkwardness. Our future research objective is to analyze how cross-modal coordination of emotional facial and vocal expressions relates to perceptions of atypicality and awkwardness of individuals with ASD.

Fourth, the sample of children with ASD in the present study had relatively high cognitive ability and language skills. Thus, the findings of the current study may not generalize to all individuals with ASD. Future research should measure face-voice coordination among individuals with ASD who are less cognitively and/or linguistically able.

In sum, the finding of the present study is that children with ASD show relatively weaker cross-modal integration of facial expression and speech productions, particularly during emotional speech. In contrast, NT children produce facial movements that are highly synchronous with their speech productions. These findings indicate that social communication difficulties in ASD may involve deficits of cross-modal integration of facial and vocal expressions during emotional speech production.

## Methods

### Participants

We recruited 16 children with ASD and 19 NT children for this study. We conducted vision screening (Snellen Chart) and hearing screening (pure tone portable audiometer) to determine that vision and hearing abilities were within normal limits. Participants were able to wear corrective lenses to participate in the task without interfering with eyetracking data collection. Inclusion criteria were language (Core Language Score on the Clinical Evaluation of Language Fundamentals, 5th Edition [CELF-5]^38^) and cognitive (Kaufman Brief Intelligence Test, 2nd Edition [K-BIT 2]^39^) standard scores above 79. Exclusion criteria were at least mild hearing deficit in one ear, genetic disorders, or other developmental or psychiatric diagnosis.

The ASD and NT groups did not differ significantly in age ($F(1,33)=0.60$, $p=0.44$), gender (Fishers Exact Test odds ratio: $5.72$, $p=0.20$), full IQ ($F(1,33)=0.0$, $p=1.0$) as measured by K-BIT 2^39^, or language ability ($F(1,33)=1.28$, $p=0.27$) as measured by CELF-5^38^. All children who self-reported an autism diagnosis completed the Autism Diagnostic Observation Schedule 2nd Edition [ADOS-2]^40^ with a research-reliable administrator to confirm ASD diagnosis. Of the 19 children with ASD, 13 completed Module 3 and 6 completed Module 4. Additionally, we determined the distribution of autistic symptoms in both groups (NT and ASD) using the Autism-Spectrum Quotient (AQ) and Social Communication Questionnaire [SCQ]^41^. The ASD and NT groups differed significantly in AQ ($F(1,30)=44.2$, $p=2.3\times 1{0}^{-7}$) and SCQ scores ($F(1,33)=114.9$, $p=2.8\times 1{0}^{-12}$). The children with ASD had significantly higher AQ ($t(29.9)=7.1$, $p=6.9\times 1{0}^{-8}$) and SCQ scores than the NT children ($t(20.6)=10.1$, $p=1.9\times 1{0}^{-9}$). AQ scores were missing for two participants. We used parent- or self-report of the AQ as appropriate based on the age cut-offs of the instrument. All AQ scores reported here were z-scored to adjust for the different scales of the different versions. All demographic data are found in Table 1.

**Table 1:** Demographic information for study participants by group. Abbreviations: IQ – intelligence quotient; CELF-5 – Core Language Score on the Clinical Evaluation of Language Fundamentals, 5th Edition; SCQ – Social Communication Questionnaire; AQ – Autism Quotient (z-score); ADOS-2 – Autism Diagnostic and Observation Schedule 2nd Edition; SA – social affect total; RRB – repetitive and restrictive behavior total.

Group	Gender	Age	IQ	CELF-5	SCQ	AQ	ADOS-2		
							SA	RRB	Overall
ASD N = 16	15 male	160$\,\pm \,$23 124-205	115$\,\pm \,$15 90-145	118$\,\pm \,$16 86-145	18.5$\,\pm \,$5.7 12-29	0.92$\,\pm \,$0.51 0-1.89	8.16$\,\pm \,$3.75 4-20	2.75$\,\pm \,$1.36 0-5	12.83$\,\pm \,$4.62 9-22
NT N = 19	13 male	154$\,\pm$25 124-202	115$\,\pm \,$15 91-145	112$\,\pm \,$14 93-147	2.7$\,\pm \,$2.7 0-12	-0.63$\,\pm \,$0.73 -1.3-1.69			

### Procedure

Participants were brought to the lab individually. Typically, we conducted standardized testing on one day and brought participants back for a second visit to complete several study tasks, including the sentence mimicry task described here. At the start of each session, we obtained informed assent from the participant and informed consent from a parent or guardian. The procedure for obtaining informed assent/consent and the study protocol were approved by the Institutional Review Board at Emerson College, Protocol #13-050-X-F-6.14. The research was performed in accordance with the guidelines and regulations of the Institutional Review Board at Emerson College.

**Figure 4:**
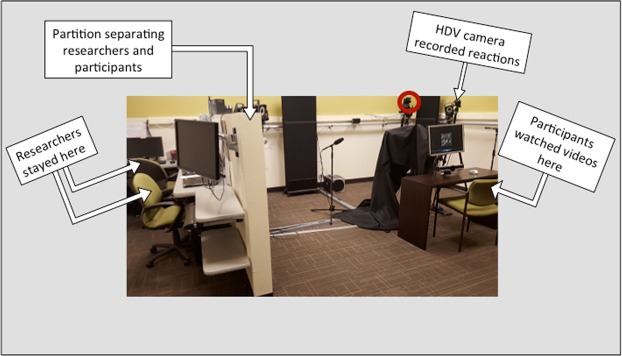
Laboratory setup where the experiment was conducted. The researcher workstation (left) was separated from the participant (right) by a partition. The video camera is circled in red. Abbreviations: HDV – high-definition video.

Participants sat in a chair with a 56 cm computer screen 60–80 cm in front of them. A video camera was positioned above and in front of the participant, but behind the computer screen (red circle in Fig. 4). We presented 16 videos of adolescent actors on the computer screen. In each video, the actor spoke a sentence with neutral emotion in the first clause and a specific emotion in the second clause (e.g., the neutral clause “I took the trash out” followed by the disgusted clause “and something gross was dripping all over me”). A range of emotions were represented, including disgust, pride, excitement, and anger (see Supplementary Material 1 for stimulus list). During the emotional clause, the stimulus actor used both voice and facial expression to convey the emotion. We recorded actors producing 112 different sentences with multiple iterations of each. Study staff chose the best iteration of each sentence based on technical criteria (focus, sound quality, etc.) and perceived clarity of the emotional expression. We then presented those best iterations of each two-phrase sentence to 34 pilot study participants in a multiple-choice task, asking them to determine the emotion (including neutral) of each phrase. We calculated the percentage of ratings confirming the target emotion and selected the stimuli with the highest agreement ratings for both the neutral and emotional phrases of a stimulus sentence (mean: 95.7%, range: 73–100%, mode: 100%), ensuring that all target emotions were represented in the final sample. We also made sure that stimuli used for this study varied in the location of emotional emphasis, so that the stressed syllable/word was located either phrase-initially, phrase-medially, or phrase-finally in order to make the mimicry task less predictable for participants.

The stimuli were presented in a pseudo-randomized sequence so that emotions, sentences, and actors did not immediately repeat. Roughly half the participants saw the sequence in reverse order. Participants were given the following instructions: “Now you are going to watch some short videos of people saying sentences. Your job is going to be to repeat the sentence as best you can. We want you to imagine yourself in the situation that the sentence describes and repeat the sentence expressing the same emotion you see the person express in the video. When it’s your turn to say the sentence look up at this video camera. It’s okay if you don’t get the words exactly right. Just try your best!” Stimuli were presented to the participant one after the other, beginning after the participant finished repeating each sentence and we allowed participants to rest between stimuli, if they requested it.

### Eyetracking

When participants sat down in front of the computer screen, we completed a five-point calibration of the SensoMotoric Instruments (SMI) RED eyetracker, which uses a sampling rate of 100 Hz, aiming for $< {1}^{\circ }$ of deviation in either axis. The RED was attached to a computer monitor mounted on a movable arm, so we could adjust the location and angle of the eye tracker and obtain optimal recording and viewing parameters for each participant. Calibration was automatic, meaning that the SMI system advanced to each of the five points as soon as participants fixated on a marker. We repeated calibration until we achieved good results, aiming for deviation of x- and y-axes of less than 1 mm. The eyetracker recorded gaze to the computer screen continuously throughout the study, marking the onset point of each stimulus sentence. We cut the gaze data at the conclusion of each sentence so as not to include gaze data to the blank screen while participants produced each sentence. We used SMI data analysis software to identify areas of interest on the upper face (including eyes, nose, upper cheeks, and forehead) and the lower face (including mouth, chin, and lower cheeks). All eyetracking data presented here are based on net dwell time to each of the areas of interest, meaning that all gaze within the area of interest is used in the calculation, not only those gazes that reach a threshold for fixation. Net dwell time is presented as a proportion of the total trial duration.

### Motion capture

**Figure 5:**
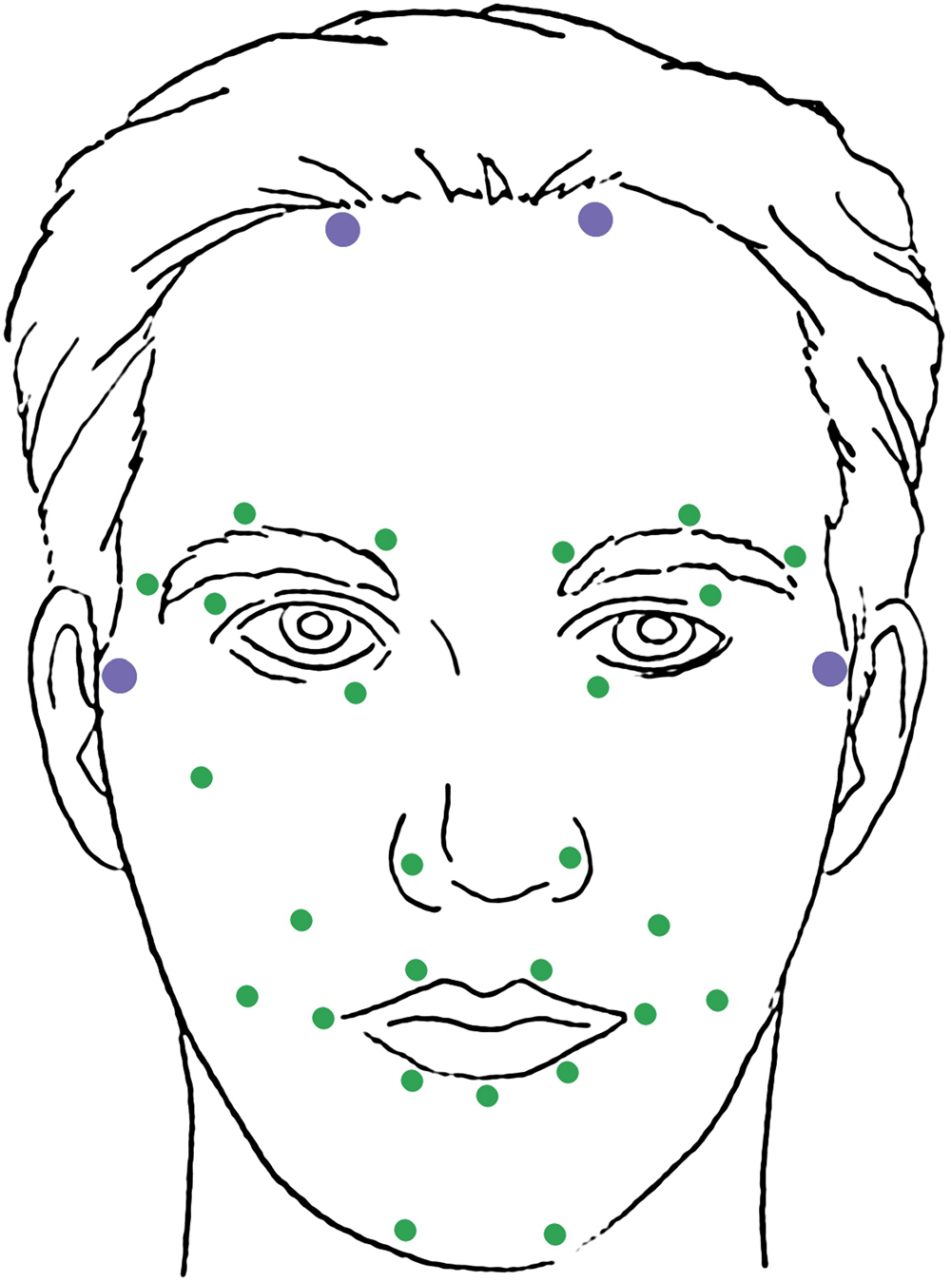
Positions of 32 reflective markers, including 4 larger stabilizer markers (purple) and 28 smaller markers (green).

Motion-capture data was captured at 100 frames per second using the VICON MX-T40 camera system (Vicon Motion Systems Ltd., Oxford, UK). We attached 28 reflective markers (4 mm diameter) to participants’ faces using defined landmarks with high movement involvement in facial expression production^42^. Four larger markers (10 mm diameter, colored red in Fig. 5) were positioned on the sides of the forehead and on both temples. These markers tracked head movements in all three planes independently of facial expressions since facial skin does not move in these areas. The x-axis indicates left-right position, the y-axis indicates inferior-superior position, and the z-axis indicates anterior-posterior position. Marker distribution was derived from the 92-marker template developed by The Digital Concepts Group, Inc. of House of Moves^43^ for the purposes of digitally animating human facial movements and expressions in the movie industry. The 92-marker template and our 32-marker derivation were based on basic facial movement patterns identified in the Facial Action Coding System^44^.

### Speech audio

The audio signal was recorded with a single-channel microphone mounted in front of the participant. The signal was sampled at 44.1 kHz. Synchronization between the audio and motion capture signals was achieved using the native software of the VICON MX-T40 camera system and audio recording devices (Vicon Motion Systems Ltd., Oxford, UK). We extracted a set of speech audio features with the openSMILE toolkit^45,46^. This included intensity, loudness, zero-crossing rate, voice probability, and fundamental frequency, which are related to voice production. Intensity is the square of the signal amplitude. Loudness is a nonlinear psychoacoustic function of intensity. Zero-crossing rate is the rate at which the audio signal crosses zero, which is high for voiceless speech segments and low otherwise. Voice probability is a measure of voicing that is close to one for voiced speech segments and close to zero for voiceless speech segments and silence. Fundamental frequency is estimated using autocorrelation and subharmonic summation. The features were extracted every 10 ms using a 25 ms Hamming window. Previous work showed that this feature set contains significant information in determining emotion states^46–48^.

### Granger causality measure of cross-modal integration

To determine the coordination between facial expression and speech production, we analyzed the facial movement (motion capture) and speech (audio) signals using Granger causality separately for each neutral and each emotional phrase. For each pair of motion capture and audio features for a given phrase, two autoregressive models of order 5 were fitted to the time series of audio features: (1) a full model with lagged values of the motion capture and audio features; (2) a reduced model with lagged values of the audio feature alone. The adjusted squared multiple correlation ${R}_{\,{\rm{audio,mocap}}\,}^{2}$ is the variance explained by the full model with audio and motion capture features as predictors. The adjusted squared multiple correlation ${R}_{\,{\rm{audio}}\,}^{2}$ is the variance explained by the reduced model with only audio features as predictors. Effect size ${R}_{\,{\rm{audio,mocap}}}^{2}-{R}_{{\rm{audio}}\,}^{2}$ was computed for the full model relative to the reduced model as a measure of the difference in variance explained between the full and reduced models. The dependent variable “cross-modal coordination” is defined as the effect size ${R}_{\,{\rm{audio,mocap}}}^{2}-{R}_{{\rm{audio}}\,}^{2},$ an indicator of Granger causality strength.

### Linear mixed effects models

We tested hypotheses using four linear mixed effects models with different specifications (see below). Each null hypothesis significance test was associated with a single coefficient of the linear mixed effects model. We used t-tests of the model coefficients to test the significance of an effect. We used the Satterthwaite approximation of the denominator degrees of freedom to obtain one p-value for each test^49^. Expected marginal means and trends were computed with R package “emmeans” in order to explore interactions^50^.

### Cross-modal coordination

The dependent variable was cross-modal coordination, a measure of Granger causality strength. Prior to analysis, we log-transformed the dependent variable for normality. The model had the following fixed effects: factor *diagnosis* with levels ASD and NT (reference level); factor *sentence* with levels emotional and neutral (reference level); factor *direction* for direction of motion capture marker movement with levels for anterior-posterior axis, superior-inferior axis, and left-right axis (reference level); covariate *uf* indicates the net dwell time to the upper face; covariate *lf* indicates the net dwell time to the lower face; covariate *age* indicates the age of the study participant. For productions of neutral sentences, covariates *uf* and *lf* corresponded to the participant watching the neutral phrase of the audio-visual stimulus. For productions of emotional sentences, covariates *uf* and *lf* corresponded to the participant watching the emotional phrase of the audio-visual stimulus. The model included all interactions between *diagnosis*, *sentence*, and *age*, all interactions between *diagnosis*, *uf*, and *age*, and all interactions between *diagnosis*, *lf*, and *age*. The model had the random intercepts for the following factors: *participant* had one level per study participant; *stimulus* had one level for each audio-visual stimulus the participant mimicked; *feature* had one level for each audio feature, indicating which audio feature is used to compute the dependent variable of Granger causality strength; *marker* had one level for each motion capture marker (excluding the four stabilizing reference markers as well as the marker on the bridge of the nose, which is used in head motion correction), indicating which motion capture marker is used to compute Granger causality strength.

### Motion capture marker/audio feature variance

The dependent variable was motion capture marker variance and audio feature variance, respectively. The model had a fixed effect for factor *diagnosis* with levels ASD and NT (reference level). The model included random intercepts for factor *participant*. The motion capture marker variance model had random intercepts for factor *marker*, and the audio feature variance model had random intercepts for factor *feature*.

### Gaze duration

The dependent variable was “gaze duration”. The model had the following fixed effects: factor *diagnosis* with levels ASD and NT (reference level); factor *sentence* with levels emotional and neutral (reference level); factor *area of interest* with levels upper face and lower face (reference level). The model included all interactions. The model included random intercepts for factor *participant* and by-*participant* random slopes for within-subjects factors *sentence* and *area of interest*.

### Statistical significance

The $\alpha =0.05$ significance threshold was Bonferroni corrected for multiple comparisons by dividing by the number of statistical tests^51^. The number of statistical tests was 36. Accordingly, the adjusted significance threshold is 0.05/36 = 1.39 $\times 1{0}^{-3}$.

## Data Availability

The raw data generated and analysed during the current study are available in the National Database for Autism Research (NDAR) repository and can be accessed by reasonable request to NDAR.

## References

[1] Baron-Cohen Simon, Wheelwright Sally, Skinner Richard, Martin Joanne, Clubley Emma (2001). Journal of Autism and Developmental Disorders.

[2] Grossman RB (2015). Judgments of social awkwardness from brief exposure to children with and without high-functioning autism. Autism.

[3] Faso DJ, Sasson NJ, Pinkham AE (2015). Evaluating posed and evoked facial expressions of emotion from adults with autism spectrum disorder. Journal of Autism and Developmental Disorders.

[4] Sasson, N.J.*et al*.Neurotypical peers are less willing to interact with those with autism based on thin slice judgments. *Sci. Reports*, **7**, 1âĂŞ10, 10.1038/srep40700 Article number: 40700. (2017). 10.1038/srep40700 PMC5286449 28145411

[5] Hubbard DJ, Faso DJ, Assmann PF, Sasson N (2017). Production and perception of emotional prosody by adults with autism spectrum disorder. Autism Research.

[6] Grossman RB, Mertens J, Zane E (2018). Perceptions of self and other: Social judgments and gaze patterns to videos of adolescents with and without ASD. Autism.

[7] Grossman RB, Edelson LR, Tager-Flusberg H (2013). Emotional facial and vocal expressions during story retelling by children and adolescents with high-functioning autism. Journal of Speech Language and Hearing Research.

[8] Nadig A, Shaw H (2012). Acoustic and perceptual measurement of expressive prosody in high-functioning autism: Increased pitch range and what it means to listeners. Journal of Autism and Developmental Disorders.

[9] Bone, D. *et al*. Spontaneous-speech acoustic-prosodic features of children with autism and the interacting psychologist. In *Proceedings of Interspeech*, 1043âĂŞ-1046 (2012).

[10] Bone, D., Black, M. P., Ramakrishna, A., Grossman, R. & Narayanan, S. Acoustic-prosodic correlates of ‘awkward’ prosody in story retellings from adolescents with autism. In *Proceedings of Interspeech*, 1616–1620 (2015). 10.21437/interspeech.2015-374 PMC12978205 41821926

[11] Grossman RB, Bemis RH, Skwerer DP, Tager-Flusberg H (2010). Lexical and affective prosody in children with high-functioning autism. Journal of Speech, Language, and Hearing Research.

[12] Peppé S, Cleland J, Gibbon F, O’Hare A, Castilla PM (2011). Expressive prosody in children with autism spectrum conditions. Journal of Neurolinguistics.

[13] Van Santen JP, Prud’Hommeaux ET, Black LM, Mitchell M (2010). Computational prosodic markers for autism. Autism.

[14] Zane E (2019). Motion-capture patterns of voluntarily mimicked dynamic facial expressions in children and adolescents with and without ASD. Journal of Autism and Developmental Disorders.

[15] Metallinou, A., Grossman, R. B. & Narayanan, S. Quantifying atypicality in affective facial expressions of children with autism spectrum disorders. In Quantifying atypicality in affective facial expressions of children with autism spectrum disorders. In *Proceedings of the IEEE International Conference on Multimedia and Expo*, 1–6, 10.1109/ICME.2013.6607640 (IEEE, 2013) . 10.1109/ICME.2013.6607640 PMC4188372 25302090

[16] Guha, T. *et al*. On quantifying facial expression-related atypicality of children with autism spectrum disorder. In *Proceedings of the IEEE International Conference on Acoustics, Speech, and Signal Processing*, 803–807, 10.1109/ICASSP.2015.7178080 (IEEE, 2015). 10.1109/ICASSP.2015.7178080 PMC4687751 26705397

[17] Guha T, Yang Z, Grossman RB, Narayanan SS (2018). A computational study of expressive facial dynamics in children with autism. IEEE Transactions on Affective Computing.

[18] Klin A, Jones W, Schultz R, Volkmar F, Cohen D (2002). Visual fixation patterns during viewing of naturalistic social situations as predictors of social competence in individuals with autism. Archives of General Psychiatry.

[19] Nakano T (2010). Atypical gaze patterns in children and adults with autism spectrum disorders dissociated from developmental changes in gaze behaviour. Proceedings of the Royal Society B: Biological Sciences.

[20] Pelphrey KA (2002). Visual scanning of faces in autism. Journal of Autism and Developmental Disorders.

[21] Tanaka JW, Sung A (2016). The “eye avoidance” hypothesis of autism face processing. Journal of Autism and Developmental Disorders.

[22] Fletcher-Watson S, Findlay JM, Leekam SR, Benson V (2008). Rapid detection of person information in a naturalistic scene. Perception.

[23] Fletcher-Watson S, Leekam SR, Benson V, Frank MC, Findlay JM (2009). Eye-movements reveal attention to social information in autism spectrum disorder. Neuropsychologia.

[24] McPartland JC, Webb SJ, Keehn B, Dawson G (2011). Patterns of visual attention to faces and objects in autism spectrum disorder. Journal of Autism and Developmental Disorders.

[25] Falck-Ytter Terje, von Hofsten Claes (2011). How special is social looking in ASD. Progress in Brain Research.

[26] Papagiannopoulou EA, Chitty KM, Hermens DF, Hickie IB, Lagopoulos J (2014). A systematic review and meta-analysis of eye-tracking studies in children with autism spectrum disorders. Social Neuroscience.

[27] Stevenson RA (2014). Multisensory temporal integration in autism spectrum disorders. Journal of Neuroscience.

[28] Golan O, Gordon I, Fichman K, Keinan G (2018). Specific patterns of emotion recognition from faces in children with ASD: Results of a cross-modal matching paradigm. Journal of Autism and Developmental Disorders.

[29] Grossman RB, Tager-Flusberg H (2012). “Who said that?” Matching of low-and high-intensity emotional prosody to facial expressions by adolescents with ASD. Journal of Autism and Developmental Disorders.

[30] de Marchena A, Eigsti I-M (2010). Conversational gestures in autism spectrum disorders: Asynchrony but not decreased frequency. Autism Research.

[31] Granger CWJ (1969). Investigating causal relations by econometric models and cross-spectral methods. Econometrica: Journal of the Econometric Society.

[32] Klin A, Lin DJ, Gorrindo P, Ramsay G, Jones W (2009). Two-year-olds with autism orient to non-social contingencies rather than biological motion. Nature.

[33] Hamilton AFdC (2013). Reflecting on the mirror neuron system in autism: A systematic review of current theories. Developmental Cognitive Neuroscience.

[34] Rizzolatti G, Fogassi L (2014). The mirror mechanism: recent findings and perspectives. Philosophical Transactions of the Royal Society B: Biological Sciences.

[35] McIntosh DN, Reichmann-Decker A, Winkielman P, Wilbarger JL (2006). When the social mirror breaks: Deficits in automatic, but not voluntary, mimicry of emotional facial expressions in autism. Developmental Science.

[36] Khalil R, Tindle R, Boraud T, Moustafa AA, Karim AA (2018). Social decision making in autism: On the impact of mirror neurons, motor control, and imitative behaviors. CNS Neuroscience & Therapeutics.

[37] Zane E, Neumeyer K, Mertens J, Chugg A, Grossman RB (2017). I think we’re alone now: Solitary social behaviors in adolescents with autism spectrum disorder. Journal of Abnormal Child Psychology.

[38] Wiig, E. H., Semel, E. & Secord, W. A. *Clinical Evaluation of Language Fundamentals* (NCS Pearson, Inc, Bloomington, MN, 2013), 5 edn.

[39] Kaufman, A. & Kaufman, N. *Manual for the Kaufman Brief Intelligence Test*, 2 edn. (American Guidance Service, Circle Pines, MN, 2004).

[40] Lord, C. *et al*. *Autism Diagnostic Observation Schedule Second Edition (ADOS-2) Manual (Part 1): Modules 1âĂŞ4*, 1 edn.(Western Psychological Services, Torrance, CA, USA, 2012).

[41] Rutter, M., Bailey, A. & Lord, C. *SCQ: The Social Communication Questionnaire*, 1 edn. (Western Psychological Services, Los Angeles, CA, USA, 2003).

[42] Trotman C-A, Faraway JJ, Silvester KT, Greenlee GM, Johnston LE (1998). Sensitivity of a method for the analysis of facial mobility: I. Vector of displacement. The Cleft Palate-Craniofacial Journal.

[43] Hauck, J. & D., J. *House of Moves High Resolution Facial Marker-set (92 Markers)*, 1 edn. (Digital Concepts Group, Inc. House of Moves, Los Angeles, CA, USA, 2007).

[44] Ekman, P. & Friesen, W. V. *Facial Action Coding System*, 1 edn. (Consulting Psychologists Press, Palo Alto, CA, 1977).

[45] Eyben, F., Weninger, F., Gross, F. & Schuller, B. Recent developments in openSMILE, the Munich open-source multimedia feature extractor. In *Proceedings of the 21st ACM International Conference on Multimedia*, 835–838, 10.1145/2502081.2502224 (ACM New York, NY, USA, 2015). .

[46] Schuller, B., Steidl, S. & Batliner, A. The INTERSPEECH 2009 Emotion Challenge. In *Proceedings of Interspeech*, 312–315 (2009).

[47] Cummins, N., Epps, J., Breakspear, M. & An investigation of depressed speech detection: Features and normalization. In Cosi, P., De Mori, R., Di Fabbrizio, G. & Pieraccini, R. (eds.) *Proceedings of Interspeech*, 2997–3000 (2011).

[48] Rehg, J. *et al*. Decoding childrenâĂŹs social behavior. In *Proceedings of the IEEE Conference on Computer Vision and Pattern Recognition*, 3414âĂŞ- 3421, 10.1109/CVPR.2013.438 (IEEE, 2013).

[49] Kuznetsova A, Brockhoff PB, Christensen RHB (2017). lmerTest package: Tests in linear mixed effects models. Journal of Statistical Software.

[50] Lenth R (2018). Package ‘lsmeans’. The American Statistician.

[51] Helberg, C. Multiple comparisons. In Salkind, N. J. (ed.) *Encyclopedia of Measurement and Statistics*, 645–648, 10.4135/9781412952644.n297 1 edn. (SAGE Publications, Inc., Thousand Oaks, CA, 2007).

